# Metabarcoding reveals that rhizospheric microbiota of *Quercus pyrenaica* is composed by a relatively small number of bacterial taxa highly abundant

**DOI:** 10.1038/s41598-018-38123-z

**Published:** 2019-02-08

**Authors:** Ana V. Lasa, Antonio J. Fernández-González, Pablo J. Villadas, Nicolás Toro, Manuel Fernández-López

**Affiliations:** Departamento de Microbiología del Suelo y Sistemas Simbióticos, Estación Experimental del Zaidín, CSIC, calle Profesor Albareda 1, 18008 Granada, Spain

## Abstract

Melojo oak (*Quercus pyrenaica* Willd.) is a key tree species of Mediterranean forests; however, these forests show an advanced stage of deterioration in the Iberian Peninsula. Plant-associated microorganisms play an essential role improving their host’s fitness, hence, a better understanding of oak rhizospheric microbiome, especially of those active members, could be the first step towards microbiome-based approaches for oak-forest improvement. Here we reported, for the first time, the diversity of total (DNA-based) and potentially active (RNA-based) bacterial communities of different melojo-oak forest formations through pyrosequencing of 16S rRNA gene amplicons. We found that potentially active bacterial communities were as rich and diverse as total bacterial communities, but different in terms of relative abundance patterns in some of the studied areas. Both core microbiomes were dominated by a relatively small percentage of OTUs, most of which showed positive correlation between both libraries. However, the uncoupling between abundance (rDNA) and potential activity (rRNA) for some taxa suggests that the most abundant taxa are not always the most active, and that low-abundance OTUs may have a strong influence on oak’s rhizospheric ecology. Thus, measurement of rRNA:rDNA ratio could be helpful in identifying major players for the development of bacterial bioinoculants.

## Introduction

Melojo oak (*Quercus pyrenaica* Willd.) is a sub-humid Mediterranean mountainous species extended from Southwestern France to the North of Morocco^1^. *Quercus pyrenaica* is less commonly found out of the southern limits of its chorology, being considered as a relict species in National and Natural Park of Sierra Nevada, in Southeast Spain^2^. This tree species has been historically managed with silvo-pastoral purposes and used in a wide variety of industries^3^, due to its wood and bark properties^1^. However, the replacement of melojo oak forests with fast-growing tree species such as *Pinus* spp. and *Eucalyptus globulus*^4^ and the increasing number and frequency of fires has led to a great habitat fragmentation and deterioration of these forests. Besides human activity, natural factors concerning acorn production^5^, the harsh summer conditions typical of the Mediterranean region^6^, herbivore defoliation and certain kinds of pests^7–9^, have impacted the natural regeneration and survival of oak plantations in a meaningful way^1^. Consequently, melojo oak forests show a worrying state of degradation nowadays^2^.

Plants harbour a wide variety of microorganisms in nearly all tissues, both inside and outside their surfaces. The portion of soil influenced by plant roots, the rhizosphere, is a niche of great microbial diversity^10^ strongly determined by plant metabolism through roots exudates. At the same time, plant-associated microorganisms, especially those found in the rhizosphere, play significant roles in plant nutrition and adaptation to different types of stress^11^. Indeed, plant-microbiome could represent an additional source of genes and functions to its host, which may expand plants’ own ability to adapt to several environmental changes^12^. Consequently, plant-associated microorganisms, specifically bacteria, might improve their host’s growth and survival, that is, the overall plant fitness. Thus, plants should be considered as a whole ‘holobiont’ instead of standalone organisms^11^.

Some authors have already demonstrated that different bacterial species can promote the growth of certain forest species’ plantlets such as *Pinus pinea*^13,14^ and even other species of *Quercus* genus, for instance *Q. ilex* subsp. *ballota*^13,15^. Rhizobacteria not only can enhance the growth of woody species but also can improve the drought tolerance of their hosts, such as *Q. coccifera* plantlets under nursery or field conditions^16,17^. Since plant fitness is a straightforward consequence of plants’ genetic background and their associated microbiome, plants should be considered as holobionts in future afforestation tasks. Indeed, better understanding of the bacterial component of the holobiont could be helpful improving the establishment and survival of *Q. pyrenaica* plantations in natural environments.

During the last years, many attempts have been made to describe the bacterial microbiome of many host plants, including *Q. pyrenaica*^18^, by means of DNA high-throughput sequencing^19–23^. However, few works have focused on those components of bacterial microbiomes that are actually active and capable of influencing hosts plant fitness. 16S rRNA gene-based surveys cannot discern between dormant prokaryotic populations, active members, dead cells and environmental DNA, whereas the short lifespan and fast turnover of RNA in soil make RNA transcripts an outstanding target for plant microbiome description analyses. Hence, RNA-based strategies are a powerful tool to assess the diversity of proteins-synthesizing prokaryotic members, which are more likely to be metabolically active at a given moment and linked to soil functions^24^. Furthermore, combining analyses of total and potentially active populations could provide a more comprehensive view of bacterial ecology and its member’s influence on forest soils. In this sense, Cobo-Díaz *et al*.^18^ characterised the functional diversity of the bacterial communities dwelling in the rhizosphere of melojo-oak by a metagenomic approach. However, these authors focus its work on the metabolic pathways related to methane, sulfur and nitrogen, and without an RNA approach of these bacterial communities, therefore the potentially active taxa of melojo oak rhizosphere remain unexplored.

Unraveling the structure of rhizospheric bacterial assemblages associated with woody species –and especially those metabolically active strains– could represent the first step towards the development of an effective bioinoculant in forest restoration biotechnology. Therefore, in this work we analysed the prokaryotic diversity of *Quercus pyrenaica* rhizosphere at three sites within the National and Natural Park of Sierra Nevada (Spain) covered with forests at different development stages. We aimed to define the core of total and potentially active prokaryotic populations inhabiting melojo oak’s rhizosphere through 16S rRNA gene amplicon pyrosequencing based on DNA and RNA-derived libraries, respectively. We also focused on those taxa with the highest ratio of the relative abundance of the RNA-based versus DNA-based (rRNA:rDNA), with the aim of identifying the main prokaryotic players of *Q. pyrenaica* rhizospheric microbiome in order to find native potential candidates as plant inoculants.

## Results

### Physicochemical properties of the soils

The soils from the study areas were sandy loam (XZF and BRF) or loam (HAF). Statistically significant differences were detected in several physicochemical parameters between sites (Supplementary Table S4). The pH was slightly acid in BRF site and close to neutrality especially in HAF mature forest; indeed, both of them were significantly different (P = 0.029). The percentage of soil organic matter in HAF forest was more than two-fold higher than that of BRF site, and both sites differed also in the percentage of available water as well as in the salinity level (P < 0.02). There were no significant differences in total nitrogen (N) proportion between any of the studied sites (Supplementary Table S4).

### General characteristics of pyrosequencing datasets and alpha-diversity indices

We obtained 147,453 and 132,096 raw reads for rDNA-based and rRNA-based libraries, which after the trimming step resulted in 109,236 and 76,310 high-quality sequences, respectively. The number of sequences extracted from each sample is summarized in Supplementary Table S1. Calculated rarefaction curves at 3% of genetic distance tended to the asymptote (Supplementary Figure S1). Furthermore, Good’s coverage values ranged from 88.8 to 95.5% for DNA samples and from 83.2 to 94.6% in the case of RNA samples, indicating that bacterial communities are well characterised and most of the rhizospheric bacterial diversity is covered in our research.

Differences in OTU richness and diversity between samples as well as between libraries (DNA and RNA) were studied after normalization by random selection of 2505 sequences per sample, which is the minimum number of sequences recorded in HAF3 for the RNA-based library. No significant differences were detected between samples or between libraries in terms of richness (Supplementary Table S1). Alpha diversity measured by Shannon index (H’) was high and similar between both libraries (P > 0.141). On the other hand, potentially active (RNA-based) bacterial communities were strikingly highly diverse; indeed, Shannon index values for XZF and BRF were as high as those recorded in the corresponding DNA surveys. However, the potentially active bacterial community showed less diversity than the whole community of the mature forest (HAF) (H’_rRNA_ = 5.82, H’_rDNA_ = 6.05, P = 0.023).

### Bacterial community composition

**Figure 1:**
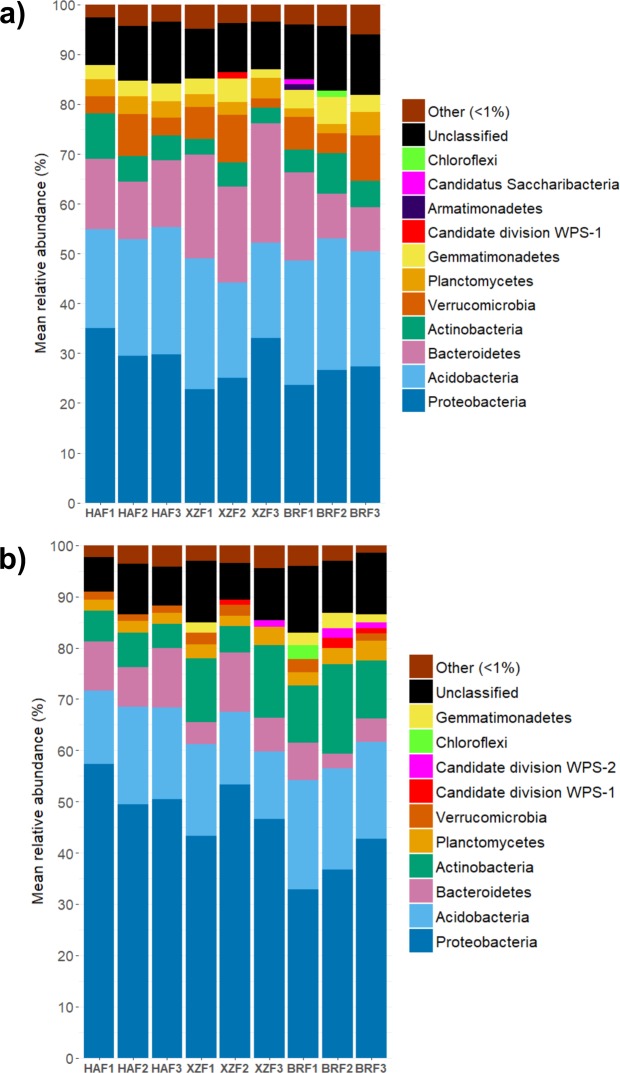
Mean relative abundance of the dominant bacterial phyla dwelling in the rhizosphere of *Q. pyrenaica* trees at DNA (**a**) and RNA (**b**) level. Rhizospheric soil samples were taken from HAF (Highest Altitudinal Forest), BRF (Burned Recovered Forest) and XZF (eXpansion Zone Forest) sites. All the phyla which accounted for less than 1% of the total sequences were grouped together in the artificial group named “Other”. Statistically significant differences in the mean relative abundances across sites and libraries are summarized in Supplementary Table S1.

By the use of trimmed, non-rarefied reads, we detected sequences related to 25 prokaryotic phyla across all samples, although only 10 were recorded in a mean relative abundance higher than 1% (Fig. 1). DNA-based libraries were mainly represented by *Proteobacteria*, *Acidobacteria*, *Bacteroidetes*, *Actinobacteria*, *Verrucomicrobia* and *Plantomycetes* members which accounted for circa 80% of the sequences. In RNA-based libraries the same phyla were predominant but with a higher relative abundance of *Proteobacteria* (Supplementary Table S2). Bacterial community composition (DNA-based libraries) was relatively stable across the three studied sites; none of the dominant phyla showed statistically significant differences in their mean relative abundance across the three locations. By contrast, the potentially active phyla from HAF and BRF sites differed at some extent; *Proteobacteria* increases in HAF with respect to BRF (52.41% and 37.45%, P < 0.04), while phylum *Gemmatimonadetes* was underrepresented in the mature forest when comparing to BRF (Fig. 1; Supplementary Table S2).

In terms of relative abundance patterns, we detected several differences between total and potentially active bacterial communities. Eight out of the 10 dominant phyla were not equally distributed among 16S rDNA and rRNA-based libraries. Proteobacterial sequences ranged from 37.45 to 52.41% in the rRNA-based library and from 25.90 to 31.45% at DNA level (Supplementary Table S2). In addition, the mean relative abundance of *Actinobacteria* was two-fold higher in the potentially active than in the total population of BRF (P = 0.04). On the other hand, in some locations, *Acidobacteria*, *Bacteroidetes*, *Verrucomicrobia*, *Planctomycetes* and *Gemmatimonadetes* were significantly less represented in rRNA-based samples with respect to the corresponding DNA libraries (Supplementary Table S2).

### Beta-diversity analyses of the bacteria communities

**Figure 2:**
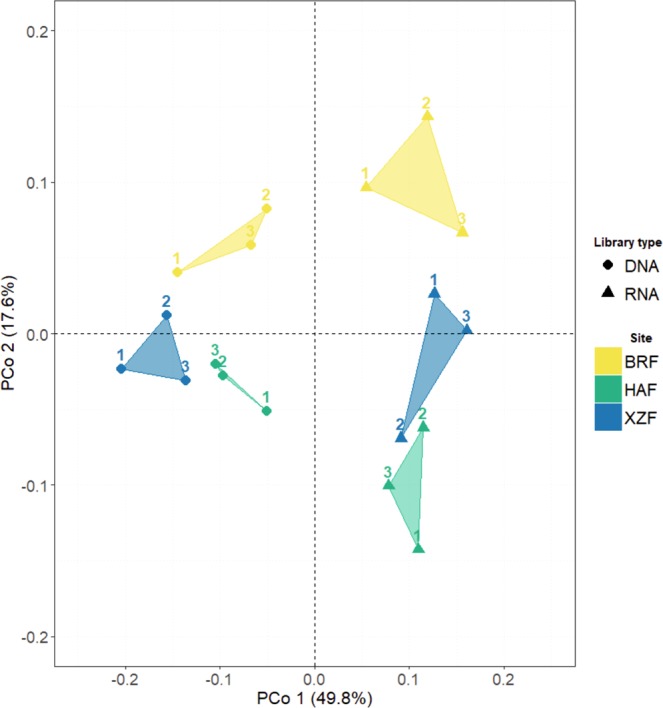
Principal Coordinate Analysis (PCoA) on weighted-UniFrac distance of prokaryotic communities from *Q. pyrenaica* Willd. rhizosphere. PCoA was constructed at OTU level, defined at 97% sequence similarity.

PCoA (Principal Coordinate Analysis) plot showed the differences found between samples in terms of the structure of bacterial communities (Fig. 2). The first PCo axis (49.8% of variance) revealed a clear distinction between total and potentially active communities. This trend was statistically supported by PERMANOVA (Permutational Analysis of Variance) analysis, which indicated that the kind of nucleic acid used for library construction was the determinant factor influencing prokaryotic communities’ composition (F = 18.388, R^2^ = 0.443, P = 0.001). Samples were separated also by the forest type, although PERMANOVA indicated that the relative contribution of this factor was significant but weaker (F = 3.940, R^2^ = 0.19, P = 0.002). DNA and RNA-based libraries were analysed separately (Supplementary Figure S2), and PERMANOVA revealed that the state of the forest contributed significantly to the differences between samples (F_rDNA_ = 2.937, R^2^_rDNA_ = 0.495, P_rDNA_ = 0.003; F_rRNA_ = 2.666; R^2^_rRNA_ = 0.470, P_rRNA_ = 0.02).

### *Quercus pyrenaica* rhizospheric core microbiomes

**Figure 3:**
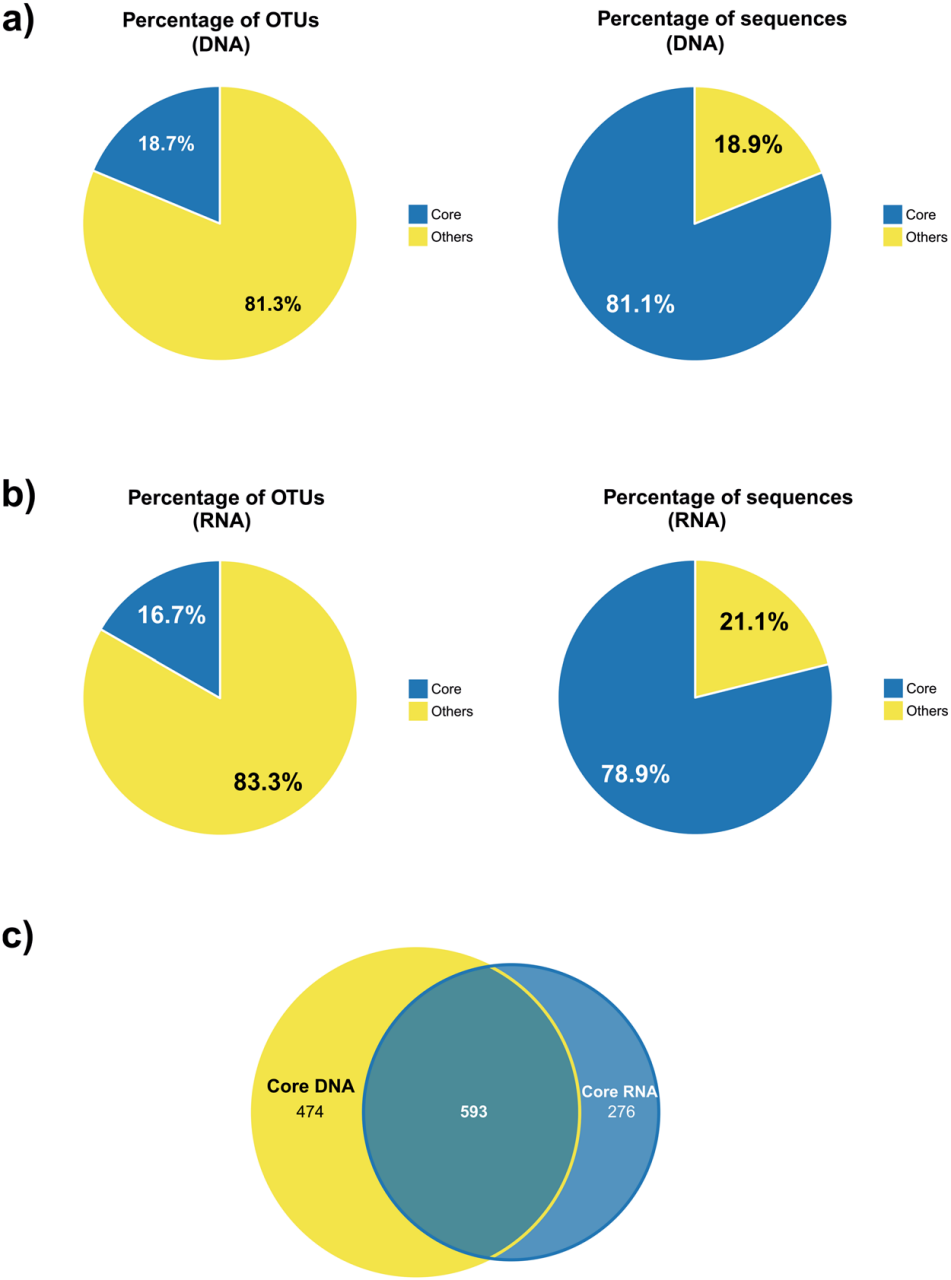
Percentage of OTUs shared between the three sampling sites (left) and sequences they accounted for (right) of DNA- (**a**) and RNA-based (**b**) rhizospheric core microbiomes. (**c**) Venn diagram showing the number of OTUs shared between both core microbiomes. The amount of OTUs of each specific microbiome is also specified.

When analysing total bacterial communities based on the DNA libraries (Fig. 3a), we detected 1067 OTUs shared between HAF, BRF and XZF samples whose abundance accounted for more than 81% of all the sequences, while they represented 18.7% of the reported OTUs (Fig. 3a). As depicted in Fig. 3b, the 3 sampling sites shared about 17% of the potentially active OTUs recorded, although they comprised almost 79% of all the rRNA-derived reads. The contribution of all site replicates to DNA- and RNA-based core microbiomes is summarized in Supplementary Fig. S3. The shared-core microbiome of melojo oak – defined by the OTUs shared by both DNA and RNA-based populations – was composed by 593 OTUs (Fig. 3c).

**Figure 4:**
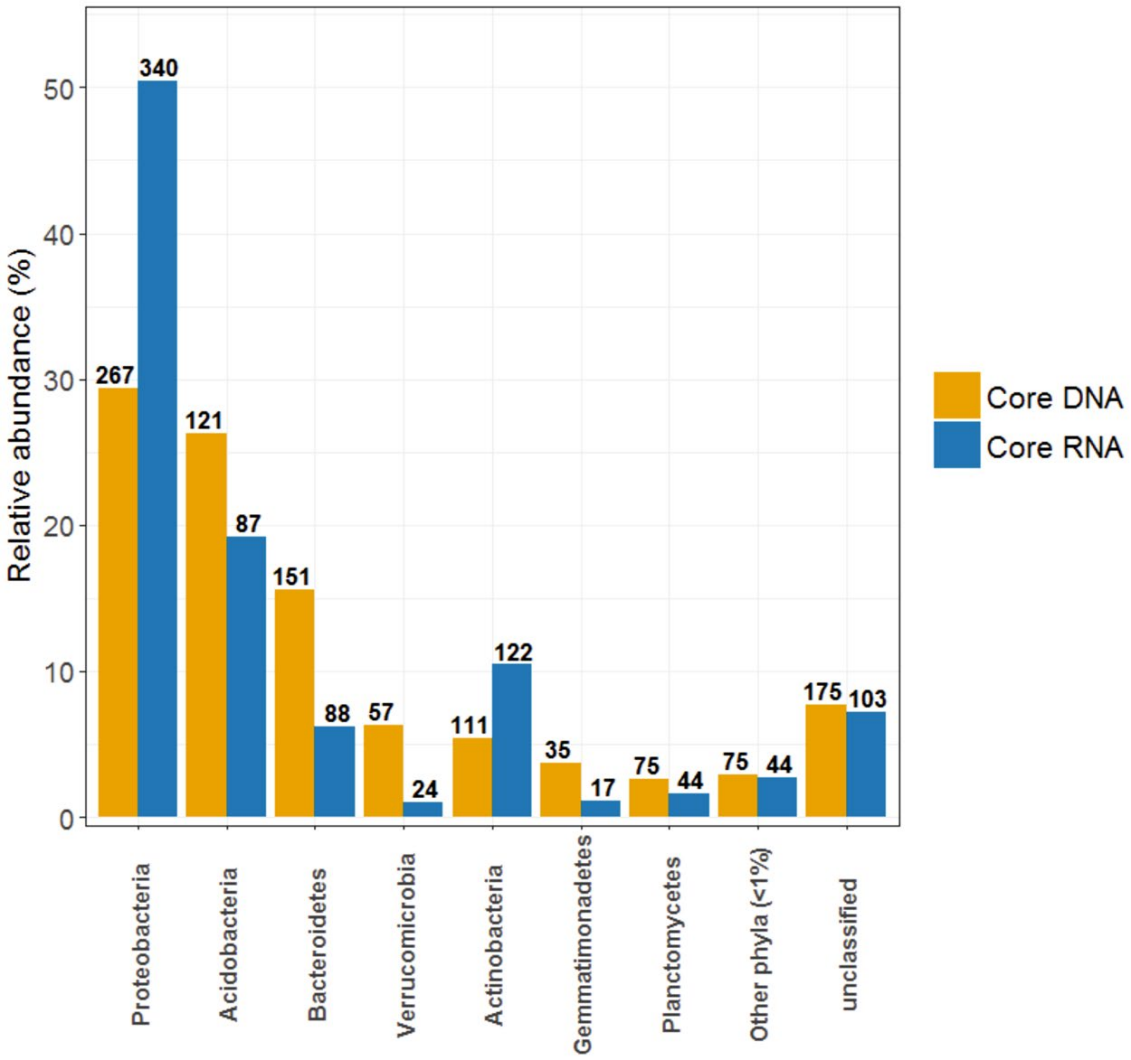
Relative abundance (%) of main phyla comprising the total (DNA-based) and potentially active (RNA-based) prokaryotic core microbiomes from melojo oak’s rhizosphere. Numbers over bars represent the OTUs number of each phylum.

We studied in depth the affiliation of those relatively few but highly represented core DNA and core RNA OTUs. There was an absolute correspondence between the composition of total and potentially active core microbiomes at phylum level, however the relative abundances of the major phyla were substantially different (Fig. 4). The core DNA microbiome was mainly dominated by *Proteobacteria, Acidobacteria, Bacteroidetes* and *Verrucomicrobia* (Fig. 4), whereas the core RNA microbiome was enriched with *Proteobacteria* (which represented almost half of the detected 16S rRNA-derived sequences), *Acidobacteria* and *Actinobacteria*. It is worth noting that it was not possible to classify roughly 7% of the recorded sequences in both cases, which indicates the high microbial diversity found in the oak rhizosphere.

**Figure 5:**
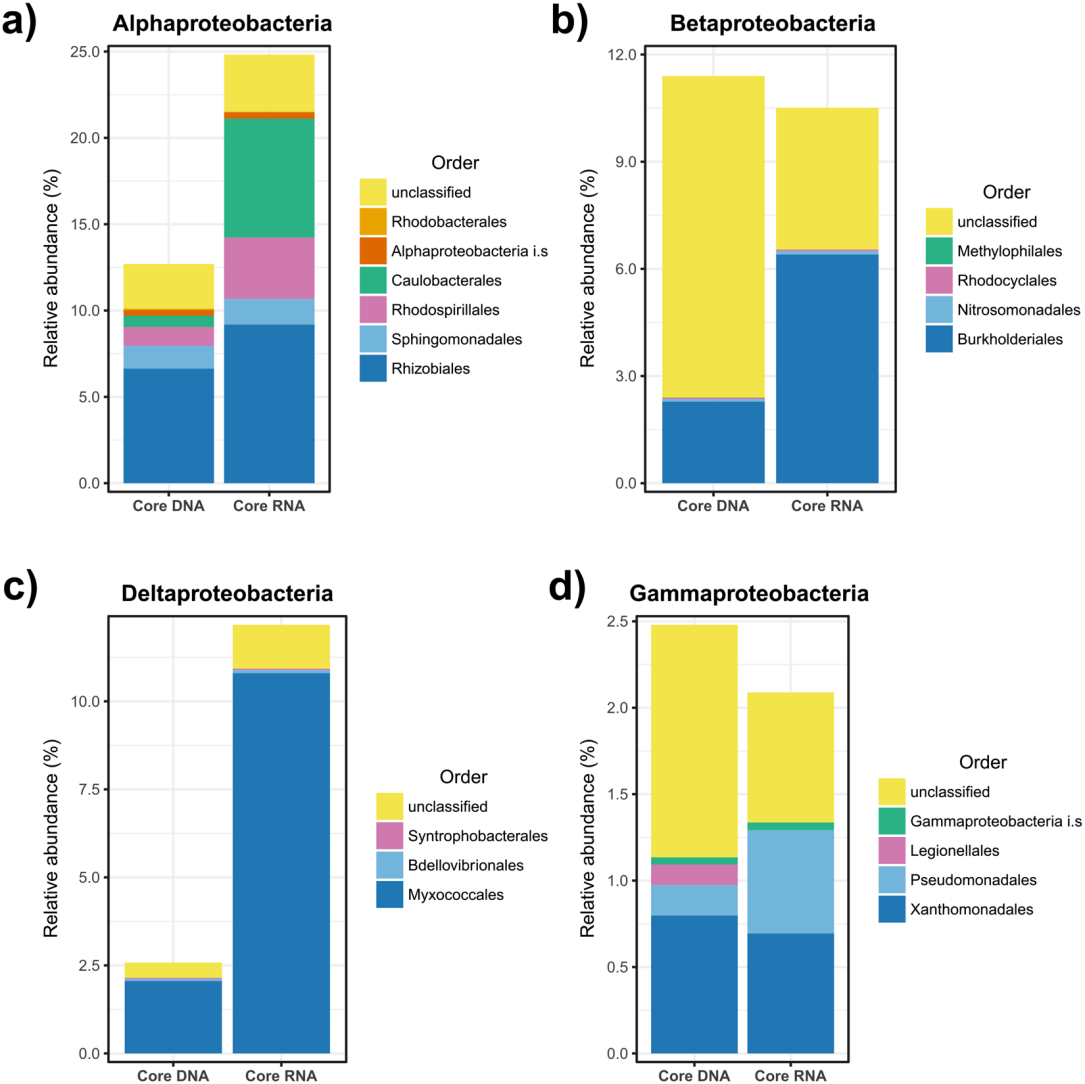
Relative abundance, expressed as percentage, of bacterial orders belonging to phylum *Proteobacteria* from DNA-based and RNA-based core microbiomes. Classes *Alphaproteobacteria* (**a**), *Betaproteobacteria* (**b**), *Deltaproteobacteria* (**c**) and *Gammaproteobacteria* (**d**) are displayed. i.s: incertae sedis.

To gain more insights into the subpopulation driving both *Q. pyrenaica* associated bacterial communities, we selected *Proteobacteria* for more detailed analyses due to its numerical dominance in both libraries, especially in the core RNA microbiome. A lower taxonomic rank analysis revealed that *Alphaproteobacteria* was mainly driven by the order *Rhizobiales* (DNA). On the other hand, potentially active community was mostly represented by *Rhizobiales* and *Caulobacterales*, which was almost depleted in the DNA library (Fig. 5a). It is important to point out that *Alphaproteobacteria* were mainly driven by *Caulobacteraceae* and an unclassified family related to order *Rhizobiales*, accounting for more than 6 and 5% of the sequences, respectively, of the potentially active core microbiome (Supplementary Table S3). By contrast, an unclassified family of the order *Rhizobiales*, and family *Bradyrhizobiaceae* were two of the most representative taxa of the core DNA-based microbiome, as shown in Supplementary Table S3.

In other classes such as *Beta*- and *Deltaproteobacteria*, we detected a strong dominance of certain orders in both studied core microbiomes. Strikingly, most of the recorded *Betaproteobacteria* from the core DNA microbiome could not be classified in a currently known order, although *Burkholderiales* was the main classified order of this class in both core DNA and core RNA microbiomes. As shown in Fig. 5c, the predominance of *Myxococcales* in the class *Deltaproteobacteria* was even stronger, both in RNA and DNA-based microbiomes. In fact, 87.45% of the deltaproteobacterial sequences derived from RNA-based library were assigned to this order, and 79.77% in the case of the DNA library.

### Relationship between abundant and potentially active OTUs of the *Q. pyrenaica* rhizospheric core microbiome

As displayed in the Venn diagram (Fig. 3c), 593 OTUs overlapped between DNA and RNA core microbiomes, with some other taxa that were library-specific (Supplementary Table S3). Among the dominant shared core OTUs (those representing more than 0.1% of the sequences in each rRNA and rDNA libraries), we observed a weak positive significant correlation between RNA- and DNA-based relative proportions (Spearman rank correlation test, ρ = 0.283, P = 0.006; Kendall rank correlation test, τ = 0.188, P = 0.008).

**Figure 6:**
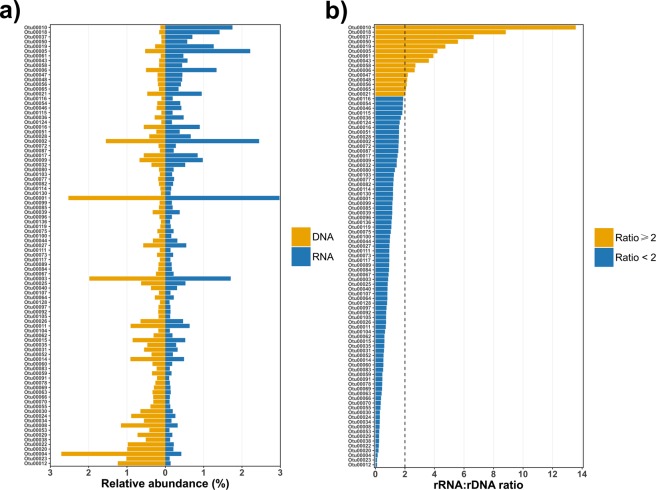
Relative abundance of the OTUs shared between the DNA- and RNA-based core microbiomes (**a**) of melojo oak and their corresponding rRNA:rDNA ratio (**b**). Only those OTUs representing more than 0.1% of the sequences in each DNA or RNA-based libraries are represented.

Two shared core OTUs closely related to *Phenylobacterium* showed a high uncoupling between abundance and potential activity, exhibiting the highest rRNA to rDNA ratio (rRNA:rDNA > 8, Fig. 6). Interestingly, the genus *Phenylobacterium* belongs to *Caulobacteraceae* family, being this disproportion in agreement with the uncoupling previously observed between the DNA and RNA-based libraries for this family (Supplementary Table S3).

## Discussion

In this study, we analysed the composition and structure of bacterial communities inhabiting *Q. pyrenaica* Willd. rhizosphere at three different sites located in the Sierra Nevada Natural and National Park: XZF, which was covered by 50-year-old isolated oak trees; BRF, a burned pine forest and later reforested in 1995 with *Q. pyrenaica* plantlets; and HAF, a natural mature forest growing at its upper altitudinal limit. The functional diversity of bacterial communities inhabiting the rhizosphere of melojo-oak was previously studied through a metagenomic approach^18^. However, these authors described the taxonomic diversity at phylum and family levels based on the phylogeny of the genes related to the methane, sulfur and nitrogen metabolisms based on DNA analyses and not on RNA. Thus, the composition of the potentially active bacterial communities associated to this woody species remains unknown up to date. In this work we analysed both total and potentially active bacterial communities at a deeper taxonomical level and with a more suitable taxonomic tool, amplicons of the 16S rRNA gene.

Although several physico-chemical properties were different among the study sites, organic matter content was the one which differed at more extent (Supplementary Table S4). The soil from mature old forest (HAF) was considerably richer in organic matter than the soil taken from the site which was burned in 1995 and reforested with oak trees. Other authors have already reported the decrease in the organic matter levels in burned forest soils in comparison with undisturbed soils^25^; however, BRF and the other unburned site (XZF) did not show significant differences in terms of this soil characteristic. On the other hand, the absence of tree canopy has expected to affect the content of organic matter especially in XZF, where just isolated single oak trees were found. However, the proportion of soil organic matter in XZF was similar to that recorded for the thickest forests, HAF and BRF. Therefore, it was not possible to elucidate which factors shaped the different organic matter levels of the studied soils. However, differences in forests state and soil characteristics helped us to better define the melojo-oak microbiome. Traditionally the microbiomes of several plants have been described through DNA-based approaches^19–23^ but this kind of surveys does not discriminate between the total and the potentially active communities. Since the rRNA-derived sequences are closely related to the content of bacterial ribosomes^26^, the strategies based on 16S rRNA gene transcripts could reflect ribosome synthesizing, individuals growing or the most adapted microbes from the total prokaryotic population, with some limitations already described^27–29^.

Both PCoA plot (Fig. 2) and PERMANOVA analyses showed that forest state has not as much influence on beta-diversity as the kind of nucleic acid has. Young trees located at XZF and BRF sites (no more than 48 and 18 years old, respectively) did not form a single cluster separated from those composing the mature forest (HAF), and neither thick forests of BRF and HAF were clearly separated from isolated single trees covering XZF site. Therefore, we were not able to distinguish any clustering pattern according to the development of forest formation. Many studies have hypothesized about the main factors driving the assemblage of the microbial communities in the rhizosphere of different plant species. As reviewed by Philippot *et al*.^30^, abiotic and biotic factors such as soil physicochemical properties and management history, climate, plant diversity, species, an even genotype and cultivars can govern rhizosphere microbiomes. Moreover, rhizosphere interactions that took place in past plant generations could be reflected in the current drivers of plant community composition. Thus, in spite of the sampling of the same tree species rhizosphere, the slight differences found in the composition of microbial communities could be due to the different forest state or forest history. Nevertheless, these slight differences could help us to define a more precise melojo-oak rhizospheric core microbiome.

It is often reported that active bacterial members are only a subset of the total bacterial population, and thus, species diversity and richness would be expected to be lower in the rRNA libraries. However, the recorded Chao-1 and diversity values for RNA-based samples were as high as those calculated for DNA libraries (except for HAF samples), especially in terms of OTU richness (Supplementary Table S1). These results are in agreement with previous analyses which compared total and potentially active prokaryotic communities in a tropical forest^31^, and in a sugar maple dominated forest^24^. Baldrian *et al*.^26^ also reported a similar diversity pattern between potentially active and total bacterial populations inhabiting *Picea abies* rhizosphere. As in these cases, our samples were collected at a time point (spring) and a natural environment where stress conditions were not expected, which could trigger some microbial taxa to reduce their metabolic activity and rRNA synthesis^32^.

Despite similar bacterial diversity and richness patterns, PCoA on weighted UniFrac distance and the subsequent PERMANOVA analysis revealed that total and potentially active bacterial communities are different. We detected several changes in the relative abundance of DNA and RNA-measured communities at the phylum level (Fig. 1); nonetheless both libraries were composed of the same bacterial phyla at each of the studied areas. Moreover, the total and potentially active phyla which dominated rhizospheric soil samples in this study, especially *Proteobacteria, Acidobacteria* and *Actinobacteria*, have been previously reported to be enriched in the rhizosphere of other *Quercus* species^33,34^. It should be pointed out that Cobo-Díaz *et al*.^18^ reported in a previous work a greater percentage of sequences related to phylum *Actinobacteria* in the rhizospheric soil of *Q. pyrenaica*, while the relative abundance of phylum *Acidobacteria* was almost three-fold lower than in our work. Since the studied areas were the same but the samplings were carried out at different experimental points, these differences could be explained by several factors not measured in this work. The main phyla detected in the rhizosphere of melojo oak trees were nearly the same as those found in the root zone of a broad range of host plants^19,22,23^, and even in some different plant tissues. Phylum *Proteobacteria* is currently considered as one of the most common phyla inhabiting not only soils^35^ and plant rhizospheres across a wide variety of plant species, but also other plant compartments^22,23^. As discussed by Coleman-Derr *et al*.^23^, these findings suggest the existence of certain conserved forces driving the structure of plant-associated bacterial populations, and these members could be regarded as a part of a generalist plant microbiome.

While each forest stage can have its own associated rhizospheric bacterial community^36^, the essential core microbiome should be common despite the specific features of each site. In this study, similar to what found Delgado-Baquerizo *et al*.^35^ at global scale, only ≈18% of the recorded OTUs were present in both DNA and RNA-based libraries at all studied sites (Fig. 3), and they accounted for a noteworthy amount of the detected DNA and RNA-derived sequences (81.1 and 78.9%, respectively). These results agree with a study of total and potentially active bacterial communities in grassland soils by Herzog *et al*.^37^; however, up to our knowledge, the current study is the first one focusing on core microbiome of a tree species. The fact that the majority of the DNA and RNA-derived sequences belong to the corresponding shared bacterial communities reflects that both, total and potentially active, core microbiomes of *Q. pyrenaica* rhizosphere are similar despite the type of forest state and the age of the trees. Vandenkoornhuyse *et al*.^11^ have recently speculated about the theoretical essential role of the core microorganisms in their hosts, suggesting that ‘accesory’ microbiome could be composed basically of more dispensable members or functions in terms of microorganism-environment interactions. The fact that just a few highly represented OTUs were shared by all the studied sites suggests that these core bacteria could play an important role along the life of the oak trees and in its resilience to different environmental conditions.

We also compared the two subsets of core DNA and core RNA OTUs in order to identify those bacterial members which were present and potentially active at the sampling time. We detected 593 core OTUs overlapping rDNA and rRNA-derived libraries (Fig. 3c), which represent 30.6% of the recorded core DNA and core RNA OTUs. Previous works also revealed that total and potentially active microbial profiles do not agree utterly, although the studied environment^38^ or the methodology they followed (16S rRNA T-RFLP fingerprinting^32^; high-density 16S rRNA microarrays^31^) were not the same as described here. Thus, we focused further analyses on those 593 OTUs shared between total and potentially active core microbiomes, since they should be the most stable part of the oak’s rhizospheric microbial community. Simulations of rRNA:rDNA ratios showed that the majority of errors committed when inferring the metabolic status of bacterial cells from these ratios were due to misclassification of active populations as dormant, while inference of active status appears generally sound^27^. It should be added that recently Papp *et al*.^39^ have come to the conclusion that even taxa with associated low rRNA:rDNA ratios can be synthesizing new nucleic acids, and therefore they are active bacteria. Although both works suggest that measurements based on rRNA:rDNA relation can underestimate the total amount of active OTUs, we focused only on those 593 OTUs with an associated value of rRNA to rDNA ratio ≥2. Therefore, our approach could reflect only a limited but accurate share of active members of *Q. pyrenaica* rhizospheric microbiome.

Historically, members of phylum *Acidobacteria* (one of the most abundant phyla in soils habitats) have been considered as K-strategists or oligotrophs in terms of lifestyle, being well adapted to low substrate availabilities and exhibiting slow growth rates^40^. The acidobacterial subgroup Gp4 was one of the most representative taxa in *Q. pyrenaica* rhizosphere (rDNA) but almost depleted in the rRNA-based library (OTUs 00004, 00020, 00022, 00038 and 00097; Fig. 6), which may fit well with the above mentioned model of K-strategists. By contrast, we did not find any uncoupling between the relative proportion of rDNA and rRNA-derived sequences for one of the most abundant taxa of melojo oak root microbiome, Acidobacteria Gp6. Naether *et al*.^41^ found higher relative abundance of Gp6 OTUs in nutrient-rich soils rather than in low-nutrient areas, and thus, they concluded that some acidobacterial populations might be more copiotrophic than often considered. Accordingly, some life history characteristics seem to be heterogeneous among all subgroups of phylum *Acidobacteria*, as we could corroborate with our results. Although the role of *Acidobacteria* Gp6 remains unclear, some of the OTUs more represented in the RNA-based library were highly abundant in the rhizosphere (Fig. 6); however, further analyses are needed to understand their role.

We also found a relatively high proportion of sequences taxonomically related to family *Bradyrhizobiaceae* (Supplementary Table S3). In our case, the abundance of this family was mainly due to genus *Bradyrhizobium*, which was previously described as one of the most abundant genera in oak rhizosphere^33^. This genus was the third most abundant in RNA library showing great adaptability to spring conditions^28^. Previous evidences support the interaction of some members of this genus with tree roots^42^.

Low-abundance bacteria, usually termed *rare taxa*, are sometimes discarded or considered unimportant in natural environments. However, bacterial abundance not always reflects each species’ contribution to the maintenance of the ecosystem; in fact, less-common populations may influence to a great extent their local environment. Rare biosphere members are essential for the maintenance of microbial alpha and beta diversity^43^ and they are thought to represent a huge reservoir of genetic and metabolic diversity that responds to different biotic or abiotic changes, contributing to the resilience of the community^44,45^. Here we observed 16 OTUs (among the most abundant species in the shared core microbiome) whose rRNA:rDNA ratio was surprisingly high, as it has been previously described in a wide variety of environments^46–49^. OTU00010 and OTU00018, closely related to *Phenylobacterium* species, showed a strikingly disproportionate percentage of rRNA-derived sequences with respect to those detected in the rDNA library (Fig. 6). These high rRNA:rDNA ratios highlight that some rare biosphere members are potentially more active than DNA-based approaches are suggesting, and thus, it points to the ecological role this genus is playing in oak’s rhizosphere. Other authors also detected several *Phenylobacterium* OTUs preferentially within the active prokaryotic communities of *P. abies* forest soils rather than in the rDNA library^26^. Although the role of the *Phenylobacterium* species has not been elucidated yet in any ecosystem, it is known that this taxon comprises some species able to degrade phenolic compounds^50^, some of which are synthesized by fungi isolated from decaying vegetation and soils^51^. It is worth mentioning that a wide variety of phenolic compounds are often found in the leaves of different species of genus *Quercus*^52^ and also in *Q. pyrenaica*’s wood^53^, so it would not be surprising that *Phenylobacterium* related species were getting involved in the degradation of the aromatic compounds resulting from oak’s decaying organic matter. However, further analyses are needed to fully understand *Phenylobacterium* ecology as a *Q. pyrenaica* core microbiome member.

Overall, the interpretation of rRNA:rDNA ratios should be done with caution due to several factors, such as different 16S rRNA gene copy number among taxa, ribosome content or growth strategy. The relation between relative abundances based on DNA and RNA libraries should not be used to report the degree of activity of bacterial taxa, but could help to classify them as active when the value of the ratio is >1, according to Steven *et al*.^27^. Therefore, we propose to additionally address specific activity of bacterial communities (e. g. by quantitative stable isotope probing^39^) when DNA and RNA surveys based in 16S rRNA gene are carried out.

## Conclusions

By combining metabarcoding techniques based on DNA and RNA, here we show that both total and ribosome-synthesizing microbiomes of *Q. pyrenaica* rhizosphere are composed by a relatively few taxa highly abundant. Although the rRNA:rDNA ratio-based approach reported here has some limitations, the uncoupling between the relative abundance of rDNA and rRNA-derived sequences could help researchers to get a more comprehensive view. Thus, some bacterial members traditionally discarded from DNA-based analyses due to its scarce relative abundance, may be strikingly highly represented in RNA-derived libraries. In this case, some members of orders *Caulobacterales*, *Burkholderiales*, *Myxococcales* and *Pseudomonadales* exhibited high rRNA:rDNA ratios, which points out that these populations should be taken into account for further analyses to assess their potential role and contribution to melojo-oak’s fitness. Our study could provide the basis for microbiome-based approaches in the field of restoration biotechnology, especially in the case of the formulation of active autochthonous bacteria-based inoculants.

## Methods

### Site description

The study area is part of Sierra Nevada National Park (SE Spain), the most southern mountain system in Europe where *Quercus pyrenaica* Willd. grows^1^. Three experimental sites covered by oak trees were selected: i) HAF, the highest altitudinal limit of the forest, where a natural mature oak forest is located, ii) XZF, the expansion zone of the forest, covered by isolated single oak trees which grew naturally after the desertion of a sorghum cropland in 1965, and iii) BRF, a pine forest burned in 1995 and subsequently afforested with melojo oak trees. The three sites have a typical Mediterranean climate with a mean annual precipitation of 555 mm, maximum temperature of 34.3 °C and minimum of −8.8 °C.

Experimental sites were sampled according to Cobo-Díaz *et al*.^25^. We selected randomly three plots per site, where we sampled the rhizosphere of three trees, each with a diameter below 15 cm at XZF and BRF sites and above 30 cm (HAF) at breast height and separated by at least 5 m. In total, we collected 27 samples, 9 from each studied site (Supplementary Figure S4 and Table S5).

### Sample collection

The rhizospheric samples were collected in the spring of 2013 according to Cobo-Díaz *et al*.^25^, and at a distance of less than 50 cm from the trunk. At each sample point and for each tree, we collected soil at depths from 5 to 25 cm, discarding the topsoil and subsequently the leaf litter and minor roots from herbaceous plants, which allowed us to sample oak rhizosphere. In order to maintain microbial community profiles, 2 g of each soil subsample were mixed with 5 ml of LifeGuard^TM^ Soil Preservation Solution (MoBio Laboratories Inc., Carlsbad, CA, USA) and immediately stored at 4 °C and at −20 °C until nucleic acid extraction, which was performed within one week of sampling. Moreover, we processed up to 1 kg of soil from each site, obtained from the roots of each tree of each plot that were pooled in order to obtain 3 replicates per site. After sieving through a 2 mm mesh, these soils were analysed for physicochemical characteristics including soil type, proportion of sand, silt, clay, available water, total nitrogen (N), organic matter and carbonates, pH, salinity and the total amount of assimilable phosphorous (P) and potassium (K). All these analyses were carried out at the Food and Agriculture Laboratory of the Andalusian regional government at Atarfe (Granada, Spain).

### Nucleic acid extraction and cDNA synthesis

RNA and DNA for each individual soil sample (in total, 27 soil samples) was co-extracted using the RNA PowerSoil® Total RNA Isolation Kit and the RNA PowerSoil® DNA Elution accessory kit (MoBio, CA, USA) respectively, following the manufacter’s recommendations. Extracted RNA was treated with DNase I (Roche, Germany) and RNase Out Recombinant Ribonuclease Inhibitor (Invitrogen, CA, USA) and the absence of DNA was confirmed by the lack of amplification of 16S rRNA gene, according to Villadas *et al*.^54^ cDNA was synthesized using random primers and SuperScript^TM^ II Reverse Transcriptase (Invitrogen, CA, USA) as recommended by the manufacturer. Double stranded cDNA was synthesized using RNase H (Roche), DNA polymerase I (Promega) and *E. coli* DNA ligase (Invitrogen, CA, USA). Blunt-end DNA was synthesized using T4 DNA polymerase (Invitrogen, CA, USA). The quantity of obtained RNA and DNA per g of soil was circa 1.0 μg and 14.8 μg, respectively as measured by Qubit 3.0 (Invitrogen, California).

### 16S rRNA gene amplicon libraries preparation and pyrosequencing

The hypervariable regions V3-V4-V5 of the 16S rRNA gene were amplified using universal primers U341F and U926R^55^, which included Roche 454-pyrosequencing A and B adapters^56,57^. PCR reaction mixtures (25 μl) contained 10 pmol of each primer, MgCl_2_ (1.8 mM), dNTPs (0.4 mM,) 1x of the corresponding Taq buffer and Taq enhancer buffer, 1 U of Taq Master (5Prime, USA) and 10 ng of DNA or cDNA as a template. The PCR conditions were the same as described by Fernández-González *et al*.^58^. Triplicate PCR reactions were performed for each DNA and RNA extracts (3 × PCR for each 27 soil samples × 2 type of templates, in total 162 PCR reactions) and the 9 PCR products coming from the same plot were subsequently pooled to reduce per-PCR variability^58^ (1 mixed sample per plot × 3 plots × 3 sites × 2 types of nucleid acids, in total 18 mixtures). The resulting composite samples were independently purified using Ultracentrifugal Filter Ultracel–100 K membranes from Amicon (Cork, Ireland) according to the manufacturer’s instructions, and QuantiFluor dsDNA System (Promega) was used to measure 16S rDNA and 16S rRNA-derived amplicon yield. For each type of nucleic acid, the 9 composite samples were sequenced with Roche’s 454 Genome Sequencer FLX pyrosequencing platform in the DNA Sequencing Service at Estación Experimental del Zaidín, Granada, Spain (Supplementary Figure S4).

### Pyrosequencing data processing and statistical analyses

Data processing was performed according to Fernández-González *et al*.^58^ using Mothur software version 1.33.3^59^, and following the protocol described in https://www.mothur.org/wiki/454_SOP. In brief, samples were demultiplexed based on the specific barcode and denoised to reduce sequencing errors. Next, a trimming step was carried out discarding low-quality sequences (those with less than 390 bp in length and/or with homopolymers longer than 8 bp). Primers and MIDs were also removed from all reads. The resulting sequences were aligned against the SILVA database^60^ and UCHIME^61^ was used to detect potential chimeras. Afterwards, high-quality prokaryotic sequences were clustered into Operational Taxonomic Units (OTUs) at 97% similarity and those OTUs represented by only a single sequence in the whole dataset (singletons) were removed. Taxonomic assignment of the remaining OTUs was performed using the Naïve Bayesian RDP (Ribosomal Database Project) Classifier and its associated database^62^. Sequences identified as chloroplasts or mitochondria were removed from the dataset.

Rarefaction curves and diversity analyses were also carried out using Mothur software. In order to eliminate the effect of sampling effort in alpha-diversity comparison analyses and to avoid bias related to different sample sizes, the number of sequences per sample was rarefied to 2505 sequences. We calculated the richness estimator Chao-1, while the diversity within each sample was evaluated using Shannon (H’) and Simpson’s reciprocal (1/D) indices. Pielou’s index (J’) was also calculated to analyse the evenness of the sampled communities.

All statistical analyses were carried out by using several packages and functions implemented in R 3.3.1^63^. The normality and homocedasticity of the data were checked by means of Shapiro-Wilk’s and Levene’s tests, respectively, before comparing soil physicochemical parameters, alpha-diversity indices (based on rarefied data) and changes in relative abundance at different taxonomical levels (non-rarefied data) among the three kinds of forest states. When data met the assumptions of normality and homocedasticity, ANOVA followed by Tukey’s HSD post-hoc test was conducted. Otherwise Kruskal Wallis and Dunn’s non-parametric tests were applied for multiple comparisons, and P-values were adjusted using the Benjamini-Hochberg method. To compare the richness and diversity indices, and the relative abundance of the main phyla between DNA and RNA-derived libraries Student’s two samples t-test (homogeneous variance) or Mann-Whitney U tests in case of parametric and non-parametric distribution, respectively.

Phyloseq package^64^ was used to calculate weighted UniFrac distances based on OTUs at 97% of genetic similarity level and to perform Principal Coordinate Analysis (PCoA) using the *UniFrac* and *ordinate* functions, respectively. A permutation test was conducted to check the homogeneity of multivariate dispersion with *betadisper* function within vegan package^65^. Since the results of this test showed that the dispersion of all sample groups were homogeneous (F = 0.786, P = 0.591), permutational ANOVA (PERMANOVA) was performed with *adonis* function included in the vegan package of R^65^ to identify factors driving sample variations. Correlations between relative abundance of DNA-based and potentially active OTUs for the shared core microbiome were examined via Kendall’s and Spearman’s correlation coefficients, which do not assume the normality of the data. The confidence level for all tests was >95% (α = 0.05).

The description of core-DNA and core-RNA microbiomes (OTU level) was performed by comparing all the samples based on rDNA or rRNA libraries, respectively, to each other, and pie charts and a Venn diagram were plotted. The latter was constructed using *draw.pairwise.venn* function in VennDiagram package^66^. Any OTU which was present in all the three studied sites and at least in the 55% of the samples was considered to be part of the corresponding core microbiome, according to Hernandez-Agreda *et al*.^67^ and also considering the cutoff selected by Delgado-Baquerizo *et al*.^35^. Afterwards, both core-DNA and core-RNA microbiomes were compared by using a Venn diagram in order to show the shared core and specific OTUs from DNA and RNA-based libraries. The rRNA:rDNA ratio was calculated for each of these shared core OTUs as the percentage of rRNA-derived sequences divided by their corresponding proportion in the DNA library. Nonetheless, a recent study^26^ has demonstrated that rRNA to rDNA ratio can misclassify active taxa as dormant and that the rate of false positive is remarkably high, therefore, here we focused only on those shared core OTUs with an associated rRNA:rDNA ratio ≥2.

## Supplementary information

Supplementary Info

## Data Availability

The datasets generated or analysed during the current study are available in the NCBI Sequence Read Archive (SRA) repository (www.ncbi.nlm.nih.gov/sra) under the BioProject accession number PRJNA379547.
