# Supplementary material for: Metabarcoding reveals that rhizospheric microbiota of Quercus pyrenaica is composed by a relatively small number of bacterial taxa highly abundant
Source: Sci Rep. 2019 Feb 8;9:1695. doi: 10.1038/s41598-018-38123-z (PMC6368570; doi:10.1038/s41598-018-38123-z)
Supplement: Supplementary file 1 — Supplementary Info [file 41598_2018_38123_MOESM1_ESM.pdf]

Metabarcoding reveals that rhizospheric microbiota of *Quercus pyrenaica* is composed by a relatively small number of bacterial taxa highly abundant

Ana V. Lasa, Antonio J. Fernández-González, Pablo J. Villadas, Nicolás Toro, Manuel Fernández-López*

Institutional address: Departamento de Microbiología del Suelo y Sistemas Simbióticos, Estación Experimental del Zaidín, CSIC, calle Profesor Albareda 1, 18008, Granada, Spain.

***Correspondence:** manuel.fernandez@eez.csic.es

Supplementary Table S1. Alpha diversity indices calculated from both DNA and RNA samples obtained from each surveyed site. HAF, High Altitudinal Forest; BRF, Burned Recovered Forest; and XZF, eXpansion Zone Forest. Diversity estimators were calculated at 97% similarity level based on 2505 sequences randomly selected per site. Shown values are means of three replicates ± standard deviations. Different Latin letters indicate statistically significant differences between sampled sites (ANOVA test, $P < 0.05$ followed by Tukey's Honest Significant Difference test), while different Greek letters show significant differences between DNA and RNA-based libraries (two sample student's t-Test, $P < 0.05$).

	DNA									RNA								
	HAF			BRF			XZF			HAF			BRF			XZF		
**Replicate**	1	2	3	1	2	3	1	2	3	1	2	3	1	2	3	1	2	3
**Raw sequences**	17702	25545	22273	7500	13502	15495	13025	19186	13225	17609	14546	4668	17021	10140	19032	19561	11927	17592
**Quality sequences**	13593	18697	17731	5180	9701	11553	9069	13952	9760	10583	8586	**2505**	10253	5823	11112	8770	7290	11388
**Good's coverage (%)**	94.69	95.53	95.36	88.80	93.13	94.12	92.66	94.70	94.56	93.75	92.52	83.15	93.03	91.69	94.11	91.90	92.09	94.58
**OTUs**	2018	2515	2322	1223	1612	1795	1665	2116	1468	1670	1580	785	2013	1164	1771	1848	1357	1662

Diversity indices after random selection of 2505 sequences

	HAF			BRF			XZF			HAF			BRF			XZF		
**Replicate**	1	2	3	1	2	3	1	2	3	1	2	3	1	2	3	1	2	3
**Number of OTUs**	877	937	888	833	818	849	861	902	787	793	841	785	1002	781	846	961	806	813
**Chao-1**	1874.64 ± 91.52 **aα**			1628.24 ± 39.80 **aα**			1683.57 ± 237.76 **aα**			1579.42 ± 168.49 **aα**			1689.60 ± 323.85 **aα**			1676.32 ± 292.21 **aα**		
**Shannon (H')**	6.05 ± 0.04 **aα**			5.96 ± 0.01 **aα**			5.94 ± 0.11 **aα**			5.82 ± 0.11 **aβ**			6.09 ± 0.20 **aα**			6.02 ± 0.13 **aα**		
**Simpson's reciprocal (1/D)**	173.92 ± 15.82 **aα**			171.69 ± 11.34 **aα**			159.00 ± 29.18 **aα**			117.26 ± 18.37 **bβ**			230.06 ± 55.25 **aα**			182.12 ± 14.74 **abα**		
**Pielou (J')**	0.89 ± 0.0041 **aα**			0.8859 ± 0.00 16**aα**			0.8816 ± 0.0108 **aα**			0.87 ± 0.0144 **aα**			0.90 ± 0.0138 **aα**			0.89 ± 0.064 **aα**		

Supplementary Table S2. Distribution of main bacterial phyla across HAF (High Altitudinal Forest), BRF (Burned Recovered Forest) and XZF (eXpansion Zone Forest) sites, in both DNA and RNA-based samples. Shown values are the mean relative abundances (%) of three replicates ± standard deviations. Different Latin letters indicate statistically significant differences between sampled sites (Kruskal-Wallis test, P < 0.05 followed by Dunn's post-hoc test), while different Greek letters show significant differences between DNA and RNA-based libraries (Mann- Whitney U test, P < 0.05).

	**DNA**			**RNA**		
	**HAF**	**BRF**	**XZF**	**HAF**	**BRF**	**XZF**
Proteobacteria	31.45 ± 3.08 **aα**	25.90 ± 1.99 **aα**	26.95 ± 4.37 **aα**	52.41 ± 4.26 **aß**	37.45 ± 4.96 **bß**	47.73 ± 5.15 **abß**
Acidobacteria	22.86 ± 2.86 **aα**	24.81 ± 1.61 **aα**	21.51 ± 3.31 **aα**	17.07 ± 3.40 **aß**	19.96 ± 1.20 **aß**	15.06 ± 2.53 **aß**
Bacteroidetes	13.03 ± 1.30 **aα**	11.81 ± 5.02 **aα**	21.35 ± 1.89 **aα**	9.67 ± 1.93 **aα**	4.94 ± 2.22 **aß**	7.53 ± 3.78 **aß**
Actinobacteria	6.43 ± 2.35 **aα**	6.04 ± 1.82 **aα**	3.71 ± 0.80 **aα**	5.79 ± 1.02 **aα**	13.27 ± 3.57 **aß**	10.55 ± 4.86 **aß**
Verrucomicrobia	5.15 ± 2.83 **aα**	6.52 ± 2.53 **aα**	5.99 ± 3.16 **aα**	1.45 ± 0.21 **aß**	1.65 ± 0.86 **aß**	1.70 ± 1.04 **aα**
Planctomycetes	3.51 ± 0.14 **aα**	2.78 ± 0.89 **aα**	3.08 ± 0.73 **aα**	2.18 ± 0.19 **aß**	3.20 ± 0.63 **aα**	2.77 ± 0.82 **aα**
Gemmatimonadetes	3.15 ± 0.34 **aα**	4.16 ± 1.47 **aα**	3.18 ± 1.20 **aα**	0.64 ± 0.25 **aß**	2.35 ± 0.73 **bß**	1.25 ± 0.58 **abα**
Candidate division WPS-2	0.55 ± 0.01 **aα**	0.68 ± 0.06 **aα**	0.42 ± 0.05 **aα**	0.69 ± 0.29 **aα**	1.20 ± 0.64 **aα**	0.88 ± 0.36 **aß**
Chloroflexi	0.62 ± 0.19 **aα**	0.98 ± 0.30 **aα**	0.71 ± 0.24 **aα**	0.58 ± 0.34 **aα**	1.27 ± 1.21 **aα**	0.45 ± 0.15 **aα**
Candidate division WPS-1	0.38 ± 0.09 **aα**	0.74 ± 0.54 **aα**	0.57 ± 0.44 **aα**	0.32 ± 0.15 **aα**	1.31 ± 0.56 **aα**	0.83 ± 0.29 **aα**

Supplementary Table S3. Relative abundance (%) of bacterial families and the corresponding orders of each proteobacterial classes of total (DNA) and potentially active (RNA) microbiomes. Taxonomic assignation was obtained with SILVA database.

Class	Order	Family	DNA	RNA
**Alphaproteobacteria**	Rhizobiales	Bradyrhizobiaceae	2.47	2.17
		Hyphomicrobiaceae	0.34	0.82
		Phyllobacteriaceae	0.31	0.36
		Rhizobiaceae	0.12	0.13
		Rhizobiales i.s	0.07	0.33
		Xanthobacteraceae	0.04	0.07
		Methylocystaceae	0.02	0.00
		Methylobacteriaceae	0.01	0.11
		Beijerinckiaceae	0.00	0.03
		Unclassified	3.26	5.17
	Sphingomonadales	Sphingomonadaceae	1.24	1.41
		Erythrobacteraceae	0.08	0.08
		Unclassified	0.00	0.00
	Rhodospirillales	Rhodospirillaceae	0.40	0.87
		Acetobacteraceae	0.21	1.03
		Unclassified	0.47	1.66
	Caulobacterales	Caulobacteraceae	0.61	6.68
		Hyphomonadaceae	0.05	0.20
		Unclassified	0.00	0.00
	Alphaproteobacteria i.s	Micropepsaceae*	0.32	0.37
		Unclassified	0.00	0.00
	Rhodobacterales	Rhodobacteraceae	0.08	0.03
		Unclassified	0.00	0.00
**Betaproteobacteria**	Burkholderiales	Comamonadaceae	0.76	1.98
		Oxalobacteraceae	0.48	0.36
		Burkholderiaceae	0.16	0.20

		Unclassified	0.88	3.86
	Nitrosomonadales	Nitrosomonadaceae	0.06	0.10
		Unclassified	0.00	0.00
	Rhodocyclales	Rhodocyclaceae	0.04	0.05
		Unclassified	0.00	0.00
	Methylophilales	Methylophilaceae	0.01	0.00
		Unclassified	0.00	0.00
**Deltaproteobacteria**	Myxococcales	Polyangiaceae	0.27	1.89
		Cystobacteraceae	0.26	1.33
		Nannocystaceae	0.00	0.05
		Haliangiaceae	0.00	0.03
		Unclassified	1.53	7.50
	Bdellovibrionales	Bdellovibrionaceae	0.06	0.08
		Unclassified	0.00	0.00
	Syntrophobacterales	Unclassified	0.03	0.04
**Gammaproteobacteria**	Xhantomonadales	Xanthomonadaceae	0.40	0.39
		Sinobacteraceae	0.31	0.24
		Unclassified	0.09	0.06
	Pseudomonadales	Pseudomonadaceae	0.18	0.60
		Unclassified	0.00	0.00
	Legionellales	Coxiellaceae	0.06	0.00
		Legionellaceae	0.06	0.00
		Unclassified	0.00	0.00
	Gammaproteobacteria i.s	Thioprofundaceae*	0.03	0.03
		Sinobacteraceae*	0.02	0.01
		Unclassified	0.00	0.00

i.s, *incertae sedis.* Families with asterisk were classified with NCBI database, since in SILVA database no adscription was obtained.

Supplementary Table S4. Physicochemical properties of HAF (Highest Altitudinal limit of the Forest), XZF (eXpansion Zone of the Forest) and BRF (Burned and Recovered Forest) soils. Mean values of three replicates and standard deviations are shown. Different letters indicate significant differences among sites (ANOVA, $p < 0.05$, Tukey's HSD post-hoc test; Kruskal-Wallis test).

**Soil properties**	**HAF**	**XZF**	**BRF**
**Type of soil**	Loam	Sandy loam	Sandy loam
**Sand (%)**	39.55 ± 4.52 **a**	59.12 ± 3.45 **b**	56.52 ± 0.98 **b**
**Silt (%)**	47.88 ± 3.57 **a**	32.58 ± 1.68 **a**	31.90 ± 0.75 **a**
**Clay (%)**	12.57 ± 1.00 **b**	8.30 ± 1.85 **a**	11.58 ± 0.77 **b**
**Available water (%)**	17.16 ± 0.83 **a**	11.57 ± 1.88 **b**	15.75 ± 2.28 **ab**
**pH (H_2O)**	6.76 ± 0.06 **a**	6.23 ± 0.42 **a**	5.97 ± 0.15 **a**
**pH (KCl)**	6.17 ± 0.06 **a**	5.60 ± 0.36 **ab**	5.27 ± 0.06 **b**
**SOM (%)**	5.99 ± 1.88 **a**	3.49 ± 0.78 **ab**	2.72 ± 0.45 **b**
**TN (%)**	0.302 ± 0.05 **a**	0.247 ± 0.12 **a**	0.168 ± 0.03 **a**
**C/N ratio**	11.20 ± 1.91 **a**	8.85 ± 2.58 **a**	9.36 ± 0.37 **a**
**Carbonates (%)**	3.03 ± 0.25 **a**	2.80 ± 0.20 **a**	1.47 ± 0.42 **b**
**Salinity (mS/cm)**	0.133 ± 0.02 **a**	0.080 ± 0.01 **b**	0.09 ± 0.02 **ab**
**Assimilable P (mg/kg)**	20.33 ± 11.59 **a**	13.00 ± 17.32 **a**	33.67 ± 8.02 **a**
**Assimilable K (mg/kg)**	433.3 ± 5.77 **a**	166.67 ± 45.09 **a**	160.0 ± 3.46 **a**

SOM, Soil Organic Matter; **TN**, Total Nitrogen

Supplementary Table S5. Characteristics of the experimental sites.

Site	Plot	Location	Altitude (masl)	Site description	Vegetation
**HAF**	1	N 36° 57’ 11.2’’ W 03° 26’ 21.0’’	1778	Natural, mature forest Highest Altitudinal limit of melojo oak Forest	*Quercus pyrenaica* Willd. forest
	2	N 36° 57’ 39.3’’ W 03° 25’ 13.6’’	1796		
	3	N 36° 57’ 55.9’’ W 03° 25’ 18.4’’	1883		
**BRF**	1	N 36° 58’ 42.7’’ W 03° 24’ 33.3’’	2017	Burned pine forest and re-afforested in 1995 with melojo oak trees	*Quercus pyrenaica* Willd. forest
	2	N 36° 58’ 40.3’’ W 03° 24’ 35.4’’	2016		
	3	N 36° 58’ 41.6’’ W 03° 24’ 39.0’’	2052		
**XZF**	1	N 36° 57’ 19.5’’ W 03° 26’ 35.0’’	1887	Expansion Zone of melojo oak Forest	*Quercus pyrenaica* Willd. isolated single trees, shrubs.
	2	N 36° 57’ 52.0’’ W 03° 25’ 49.3’’	1972		
	3	N 36° 58’ 05.9’’ W 03° 25’ 18.9’’	1975		

masl, meters above the sea level

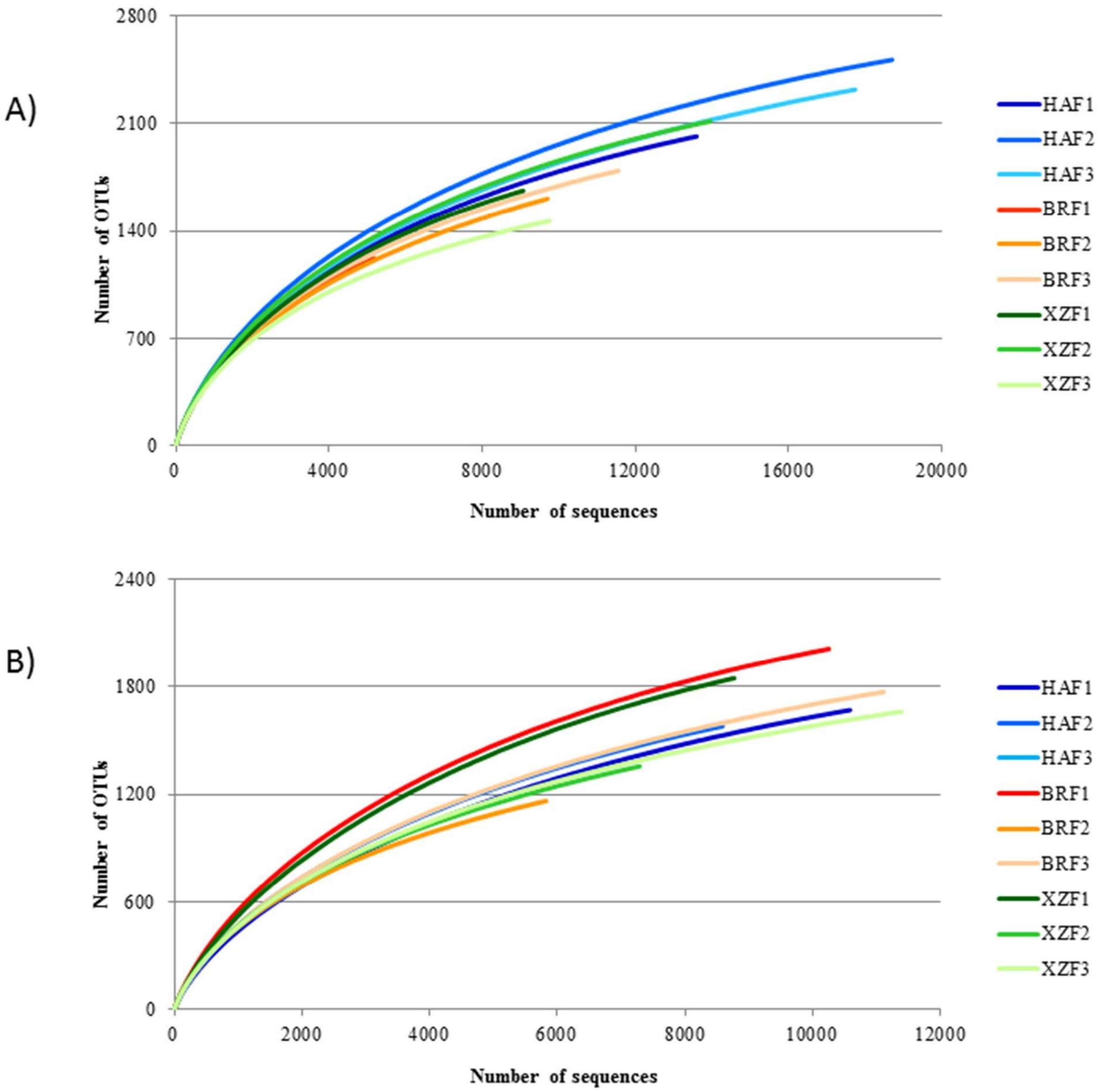

Supplementary Figure S1. Rarefaction curves calculated at OTU level (97% sequence similarity) for DNA **(A)** and RNA-based libraries **(B)**. Sampling sites correspond to HAF, High Altitudinal Forest; XZF, eXpansion Zone Forest; and BRF, Burned Recovered Forest.

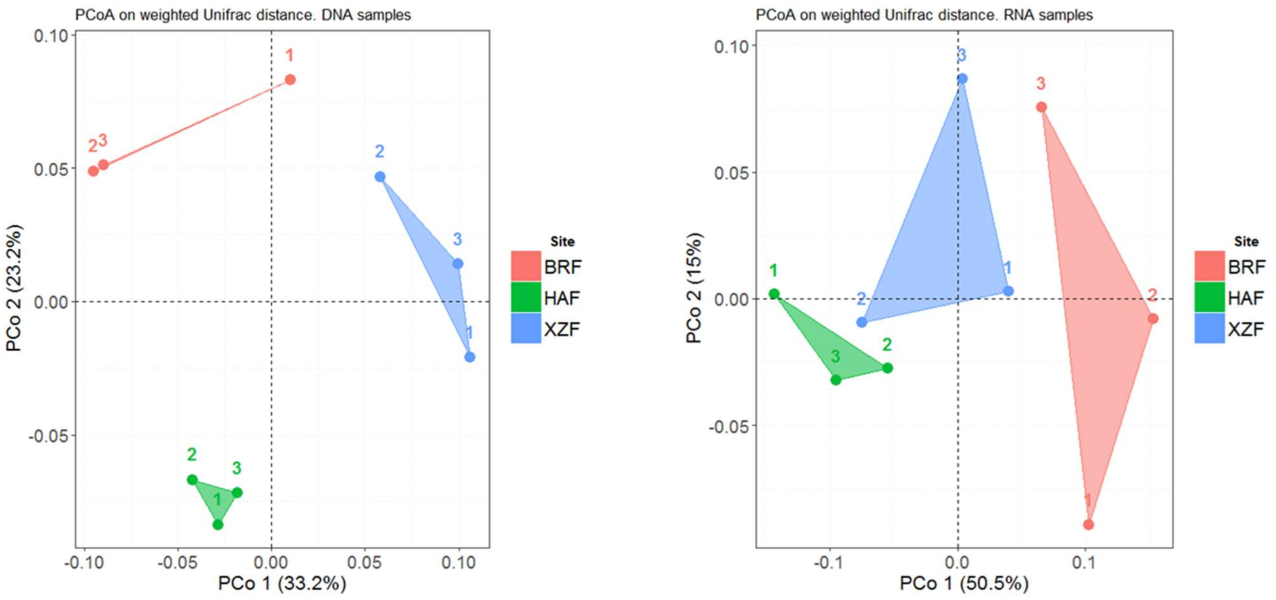

Supplementary Figure S2. Principal Coordinate Analysis (PCoA) of the DNA and RNA samples on weighted-UniFrac distance of prokaryotic communities from *Q. pyrenaica* Willd. rhizosphere. Sampling sites correspond to HAF, High Altitudinal Forest; XZF, eXpansion Zone Forest; and BRF, Burned Recovered Forest.

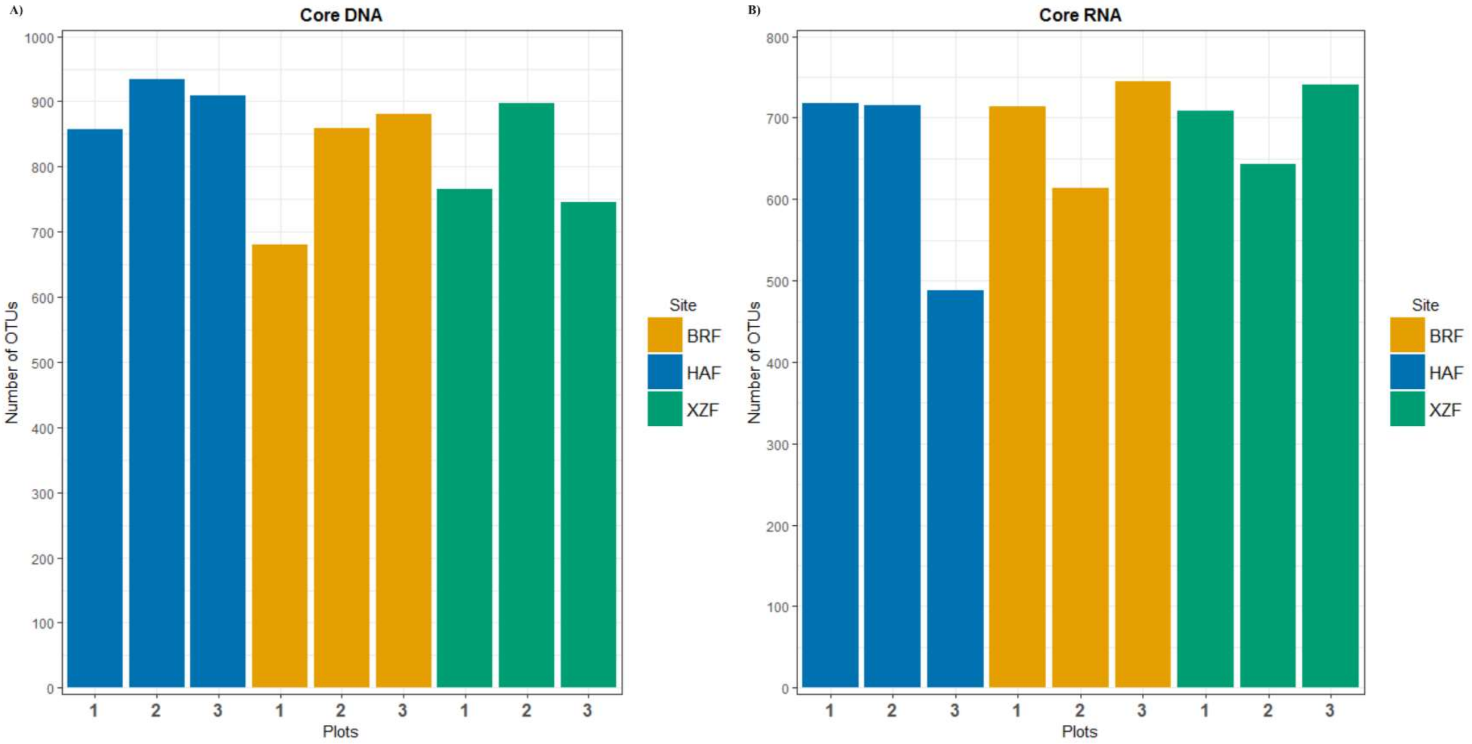

Supplementary Figure S3. Number of OTUs per plot which are part of the DNA **(A)** and RNA-based **(B)** bacterial microbiomes. Sampling sites correspond to HAF, High Altitudinal Forest; XZF, eXpansion Zone Forest; and BRF, Burned Recovered Forest.

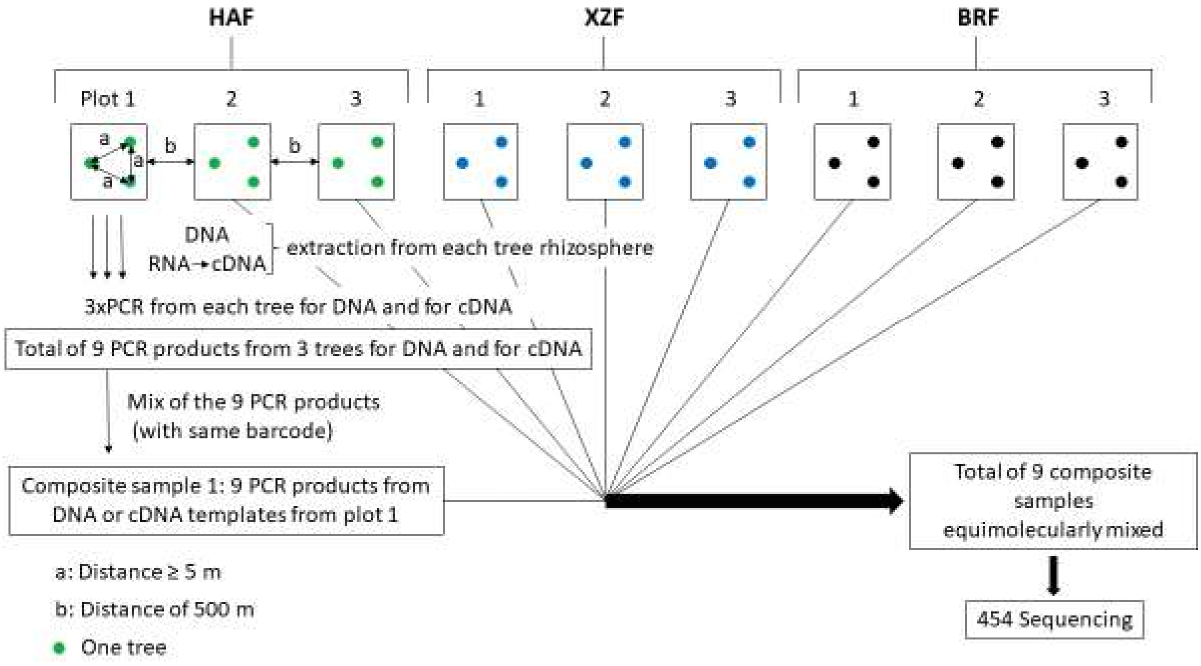

Supplementary Figure S4. Sampling scheme. Three different sites were selected (HAF, High Altitudinal Forest; XZF, eXpansion Zone Forest and BRF, Burned Recovered Forest) and in each one, three plots were delimited (3 replicates per site, n=3). In each plot, samples from the rhizosphere of three *Q. pyrenaica* trees were taken, and finally 9 composite samples were obtained for pyrosequencing.
